# Molecular Insights into the Potential Toxicological Interaction of 2-Mercaptothiazoline with the Antioxidant Enzyme—Catalase

**DOI:** 10.3390/ijms17081330

**Published:** 2016-08-16

**Authors:** Zhenxing Huang, Ming Huang, Chenyu Mi, Tao Wang, Dong Chen, Yue Teng

**Affiliations:** 1 School of Environment and Civil Engineering, Jiangnan University, Wuxi 214122, China; biogashuang@jiangnan.edu.cn (Z.H.); huangmingjn@hotmail.com (M.H.); michenyujn@hotmail.com (C.M.); wangtaojn@hotmail.com (T.W.); chendongjnedu@hotmail.com (D.C.); 2 Jiangsu Key Laboratory of Anaerobic Biotechnology, Jiangnan University, Wuxi 214122, China; 3 Jiangsu Collaborative Innovation Center of Technology and Material of Water Treatment, Suzhou 215009, China

**Keywords:** 2-mercaptothiazoline, catalase, spectroscopic technique, molecular docking, toxicity, protein conformation

## Abstract

2-mercaptothiazoline (2-MT) is widely used in many industrial fields, but its residue is potentially harmful to the environment. In this study, to evaluate the biological toxicity of 2-MT at protein level, the interaction between 2-MT and the pivotal antioxidant enzyme—catalase (CAT) was investigated using multiple spectroscopic techniques and molecular modeling. The results indicated that the CAT fluorescence quenching caused by 2-MT should be dominated by a static quenching mechanism through formation of a 2-MT/CAT complex. Furthermore, the identifications of the binding constant, binding forces, and the number of binding sites demonstrated that 2-MT could spontaneously interact with CAT at one binding site mainly via Van der Waals’ forces and hydrogen bonding. Based on the molecular docking simulation and conformation dynamic characterization, it was found that 2-MT could bind into the junctional region of CAT subdomains and that the binding site was close to enzyme active sites, which induced secondary structural and micro-environmental changes in CAT. The experiments on 2-MT toxicity verified that 2-MT significantly inhibited CAT activity via its molecular interaction, where 2-MT concentration and exposure time both affected the inhibitory action. Therefore, the present investigation provides useful information for understanding the toxicological mechanism of 2-MT at the molecular level.

## 1. Introduction

Catalase (CAT, hydrogen peroxide oxidoreductase; EC.1.11.1.6) is a common enzyme found in nearly all living organisms exposed to oxygen, which plays a pivotal role in protecting cells from oxidative injuries by catalyzing the detoxification of hydrogen peroxide to oxygen and water (2H_2_O_2_ → O_2_↑ + 2H_2_O) [1,2]. Recently, there has been growing evidence that CAT is a major factor against various pathological conditions such as diabetes, inflammation, sickle cell disease, and cancer [3,4,5,6]. However, the intake of exogenous environmental chemicals (e.g., organic pollutants, heavy metals, nanomaterials) is likely to trigger the destruction of protein conformation and to further suppress the catalytic activity of the enzyme [7,8,9]. Hence, as an important antioxidant enzyme, the toxic effects of contaminants on CAT as well as their action mechanisms should be intensively investigated in vivo and in vitro.

**Scheme 1:**
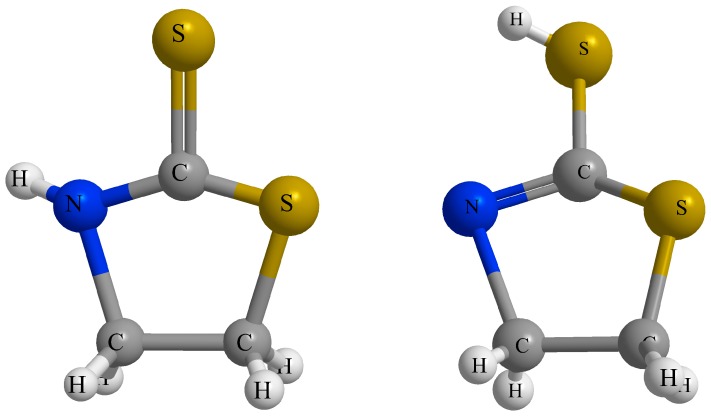
2-Mercaptothiazoline (2-MT) tautomers: thione form (**left**) and thiol form (**right**). The balls of white, gray, yellow and blue respectively represent the atoms of hydrogen (H), carbon (C), sulfur (S) and nitrogen (N), respectively.

2-Mercaptothiazoline (2-MT) is a heterocyclic organic compound with two tautomeric forms including a thione form (1,3-thiazolidine-2-thione) and a thiol form (2-thiazoline-2-thiol) [10] (Scheme 1), and 1,3-thiazolidine-2-thione is the predominant form in aqueous solution [11]. Due to its electron donor properties derived from –(NH)–(C=S)– group, 2-MT has been widely used in many fields [12]. For instance, 2-MT has been applied to synthesize brightening and stabilization agents for the printed wiring board industry as well as the photography industry [13]. In addition, based on its adsorption/complexation behaviors on the surface of metal products, 2-MT is commonly used as an organic inhibitor against corrosion and diffusion by forming metal–chelate Schiff base complexes [14]. In the environmental protection field, chemically modified 2-MT can be used to remove heavy metal ions, such as Hg(II) ions, from water solutions [15]. 2-MT derivatives are also used in the medical industry as potent anti-thyroid drugs [16]. However, 2-MT is resistant to natural and biological degradation, and thus its extensive use can result in significant residue accumulation in the environment [17]. Many studies have reported that 2-MT residue can be detected in wastewater treatment plants, surface water, as well as soils, and it is considered to be a typical organic contaminant [18,19].

2-Mercaptothiazoline is classified as a harmful organic compound, according to its Material Safety Data Sheet. Hence, the potential risk of 2-MT exposure to organisms should be investigated. Teng et al. demonstrated that 2-MT could bind into site II (subdomain IIIA) of bovine serum albumin, and provided valuable information for understanding the effects of 2-MT on the transportation of substances in the blood [20]. In addition, Thomes et al. reported that 2-MT could inhibit the activities of horseradish peroxidase, lactoperoxidase, and thyroid peroxidase [21]. Nevertheless, the current knowledge about the toxicological mechanisms of 2-MT is still severely lacking. In addition, to the best of our knowledge, there are no studies that focus on elucidating the biological toxicity of 2-MT on antioxidant enzymes at the molecular level.

Given the considerations above, in the present study, the potential toxicological interaction of 2-MT with the pivotal antioxidant enzyme—catalase (CAT) was investigated by multiple spectroscopic techniques and molecular modeling. The quenching mechanism as well as the relevant binding parameters, including the number of binding sites, binding constant, and binding forces were identified. Moreover, the interaction behaviors were further elucidated by molecular docking simulation and conformation dynamic characterization. Finally, the potential inhibitory effects of 2-MT on CAT activity caused by their interaction were verified by confirmatory experiments.

## 2. Results and Discussion

### 2.1. Characterization of the Binding Interaction between 2-MT and CAT by Fluorescence Analysis

#### 2.1.1. Fluorescence Quenching Mechanism

**Figure 1:**
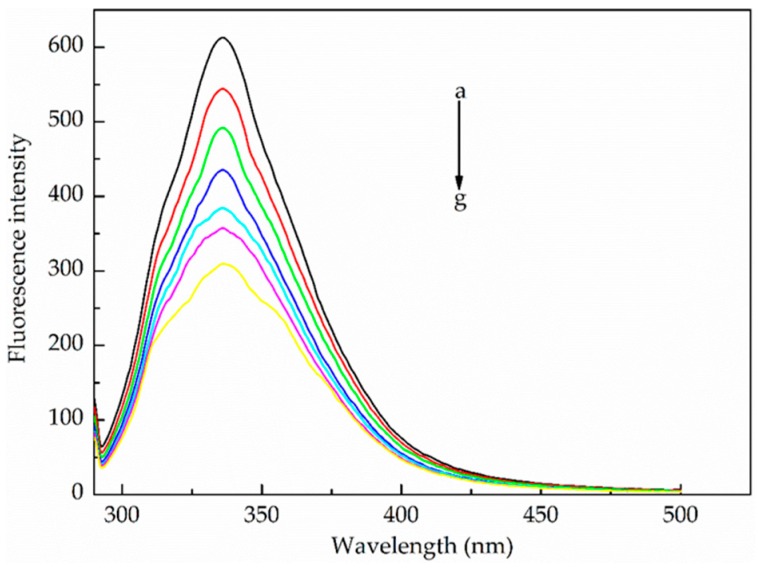
Fluorescence emission spectra of catalase (CAT) with different doses of 2-MT. Conditions: CAT: 1.0 × 10^−7^ mol·L^−1^, 2-MT: 0 mol·L^−1^ (a); 1 × 10^−5^ mol·L^−1^ (b); 2 × 10^−5^ mol·L^−1^ (c); 3 × 10^−5^ mol·L^−1^ (d); 4 × 10^−5^ mol·L^−1^ (e); 5 × 10^−5^ mol·L^−1^ (f); 6 × 10^−5^ mol·L^−1^ (g); pH 7.4; *T* = 291 K.

Fluorescence spectroscopy is a favorable technique for exploring molecular interactions since it’s has high sensitivity [22]. Fluorescence methods have been widely used to investigate the interaction between ligands and proteins, and can provide information about the quenching mechanism, binding constants and binding sites [23,24]. The fluorescence emission spectra of CAT with various doses of 2-MT additions are shown in Figure 1. The fluorescence intensity of CAT was remarkably reduced as the 2-MT concentration increased. It is known that tryptophan and tyrosine residues are mainly responsible for protein fluorescence, and the fluorescence intensity is associated with the protein structure. Thus, the CAT fluorescence quenching suggests that 2-MT could bind into CAT and potentially alter its molecular structure.

Quenching mechanisms are usually classified into either dynamic or static quenching. Since a higher temperature can result in a larger diffusion coefficient, the dynamic quenching constant should increase with increasing temperature. On the other hand, raising the temperature tends to weaken the complex stability, which can reduce the static quenching constant [25]. Hence, to better understand the mechanism of CAT fluorescence quenching caused by 2-MT, the fluorescence spectra at different temperatures were analyzed according to the Stern-Volmer equation [26].

(1) $$\frac{F_{0}}{F}=1+K_{\text{sv}}\left[Q\right]=1+k_{q}\tau_{0}\left[Q\right]$$

where *F* and *F*_0_ respectively represent the fluorescence intensities with and without the quencher, [*Q*] is the quencher concentration, τ_0_ is the average fluorescence lifetime in the absence of quencher (τ_0_ = 10^−8^ s [27]), *k*_q_ is the quenching rate constant of the biological macromolecule, and *K*_SV_ is the Stern-Volmer quenching constant.

**Figure 2:**
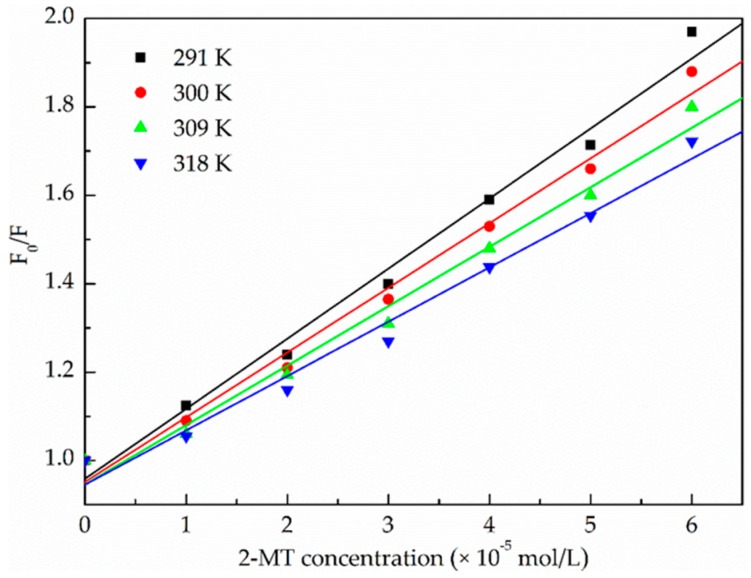
Stern–Volmer linear regression analysis for CAT fluorescence quenching caused by 2-MT under different temperatures (291 K, 300 K, 309 K, and 318 K).

Figure 2 showed the fluorescence intensity data that were analyzed based on *F*_0_/*F* versus [*Q*] at different temperatures (291 K, 300 K, 309 K, and 318 K). Subsequently, *K*_SV_ was determined using Equation (1) by a linear regression of F_0_/F against [*Q*]. As shown in Table 1, the *K*_SV_ values reduced from 1.59 × 10^4^ to 1.23 × 10^4^ L·mol^−1^·s^−1^ with the increasing temperature. For dynamic quenching, the maximum quenching rate constant (*k*_q_) is 2.0 × 10^10^ L∙mol^−1^·s^−1^ [28]. However, the *k*_q_ values under various temperatures were much greater than 2.0 × 10^10^ L∙mol^−1^·s^−1^ in this study (Table 1). Therefore, according to the results above, it could indicate that the CAT fluorescence quenching caused by 2-MT is dominated by the static quenching mechanism, through formation of the 2-MT/CAT complex, rather than dynamic quenching.

#### 2.1.2. Binding Parameters Determination

When small molecules bind independently to equivalent sites on a macromolecule via a static fluorescence quenching mechanism, the relevant parameters including the number of binding sites (*n*) and the binding constant can be determined based on the following equation [29,30].

(2) $$\text{lg}\frac{\left(F_{0}-F\right)}{F}=\text{lg}K_{a}+n\text{lg}\left[Q\right]$$

where *F*_0_, *F*, and [*Q*] are the same as in Equation (1), *K*_a_ is the binding constant and *n* is the number of binding sites. Using a linear fit of lg[(*F*_0_ – *F*)/*F*] versus lg[2-MT] under different temperatures (291 K, 300 K, 309 K and 318 K), the corresponding values of *K*_a_ and *n* can be determined from the slope and intercept. As shown in Table 2, the values of *n* were approximately 1, which implied that there should be only one site for 2-MT to bind to CAT. Moreover, the values of *K*_a_ reached an order of magnitude of 10^3^ to 10^4^, thereby indicating a strong interaction between 2-MT and CAT. Namely, even at a low concentration in cells, 2-MT can still easily bind with CAT.

#### 2.1.3. Identification of Binding Forces

Non-covalent intermolecular forces primarily cover Van der Waals’ forces, hydrogen bonding, electrostatic forces, and hydrophobic interactions, which can be identified by the calculation of thermodynamic parameters [31]. For moderate temperature change under a constant pressure, the enthalpy change (Δ*H*°) of a specific reaction can be regarded as a constant [32]. Thus, the values of Δ*H*° and Δ*S*° can be approximately calculated by the linear fit of ln*K* versus 1/*T* based on the Van’t Hoff equation (Equation (3)) [33]. Meanwhile, the free-energy change (Δ*G*°) under different temperatures can be acquired according to the classic thermodynamic equation (Equation (4)).

(3) $$\text{ln}K=-\frac{{\Delta H}^{{}^{\circ}}}{RT}+\frac{{\Delta S}^{{}^{\circ}}}{R}$$

(4) $$\Delta G^{{}^{\circ}}=\Delta H^{{}^{\circ}}-T\Delta S^{{}^{\circ}}$$

where *K* is analogous to the binding constant (*K*_a_) in Equation (2) at a specific temperature (291 K, 300 K, 309 K, and 318 K in this study). *R* is the universal gas constant (8.314 J·mol^−1^·K^−1^).

**Figure 3:**
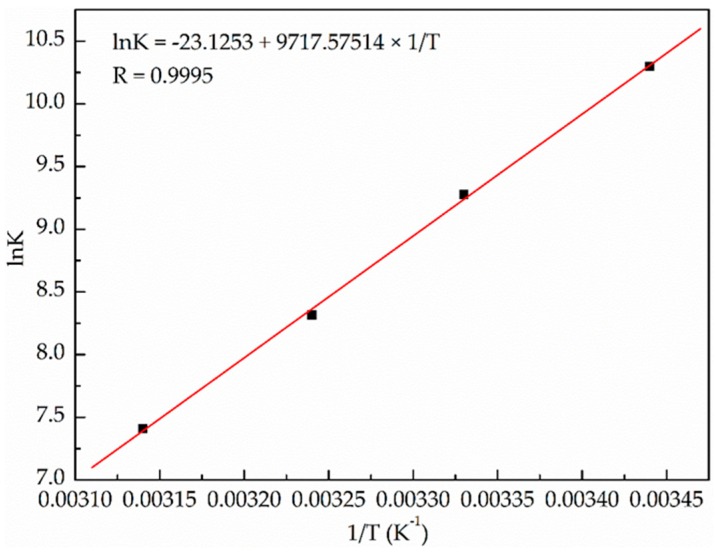
Van’t Hoff linear regression analysis for the interaction of 2-MT with CAT.

The linear regression of ln *K* versus 1/*T* for the interaction between 2-MT and CAT is shown in Figure 3. The Δ*H*° and Δ*S*° were obtained from the slope and intercept, listed in Table 2. The thermodynamic laws were used to evaluate the types of non-covalent forces: If Δ*H*° < 0, Δ*S*° < 0, the intermolecular interactions are dominated by Van der Waals’ force and hydrogen bonding; If Δ*H*° > 0, Δ*S*° > 0, hydrophobic interactions play a pivotal role in the binding reaction; If Δ*H*° < 0, Δ*S*° > 0, electrostatic interaction is the dominant force [33]. In this study, the values of Δ*H*° (−80.79 kJ·mol^−1^) and Δ*S*° (−192.26 J·mol^−1^·K^−1^) were both negative, implying that Van der Waals’ forces and hydrogen bonding are primarily responsible for the interaction between 2-MT and CAT. In addition, the negative Δ*G*° calculated from Equation (4) indicated that the interaction process should be spontaneous. Taken together, the results demonstrate that 2-MT could spontaneously bind to CAT at one binding site to form the 2-MT/CAT complex, mainly through the use of Van der Waals’ forces and hydrogen bonding.

### 2.2. Molecular Docking Simulation

**Figure 4:**
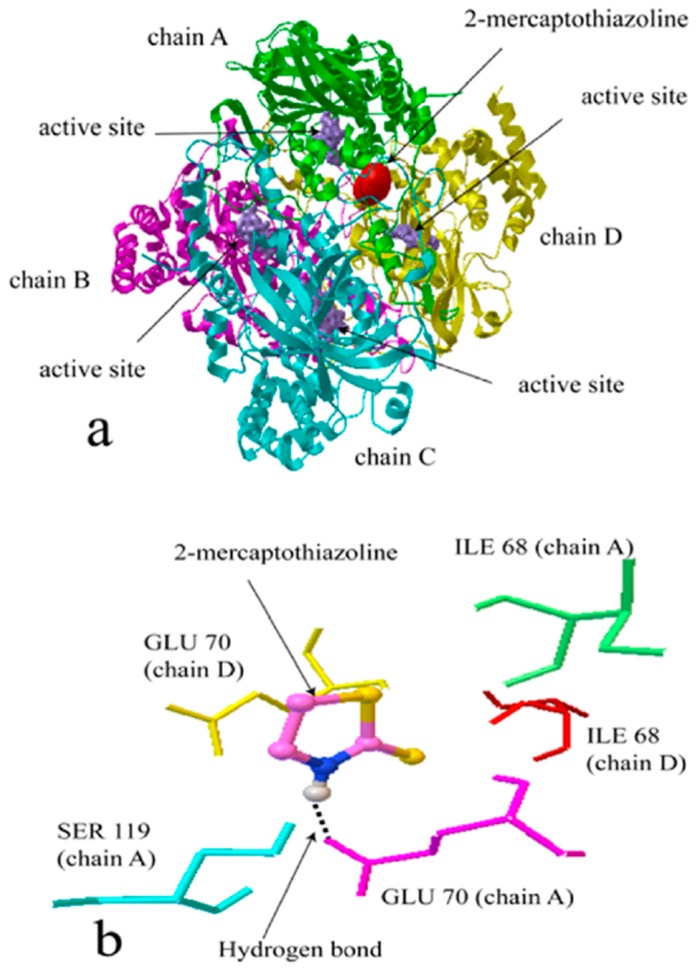
Molecular docking for the interaction between CAT and 2-MT. (**a**) Binding site of 2-MT to CAT; (**b**) Detailed illustration of the binding between CAT and 2-MT.

In this study, the molecular docking simulation was performed using AutoDock 4.2 to further characterize the interaction behaviors between 2-MT and CAT. The CAT model was obtained from the Protein Data Bank (PDB Code: 1TGU). As the predominant tautomeric form in aqueous solution, the thione form (1,3-thiazolidine-2-thione) was selected as the ligand for molecular docking, and its 3D structure was downloaded from the ZINC Database (http://zinc.docking.org/). The binding stereostructure with the lowest energy state is shown in Figure 4, which represents the optimum binding mode and the most likely binding sites. It was found that 2-MT could bind to the junctional region of the CAT subdomains (Figure 4a), and the binding site was close to the CAT active sites on chain A and chain D. The detailed docking results (Figure 4b) further revealed that the amino acid residues used to link the binding site were composed of ILE 68 (chain A), GLU 70 (chain A), SER 119 (chain A), ILE 68 (chain D), and GLU 70 (chain D). The molecular docking also indicated that the interaction between 2-MT and CAT was dominated by Van der Waals’ forces and hydrogen bonding, which was in accordance with the fluorescence analysis results above (Section 2.1.3). Figure 4b illustrates that the hydrogen bond exists between the hydrogen atoms (HN) of 2-MT and the oxygen atom (OE1) of GLU 70 (chain A).

### 2.3. CAT Conformation Dynamic Characterization

#### 2.3.1. UV–Vis Absorption Spectroscopy

**Figure 5:**
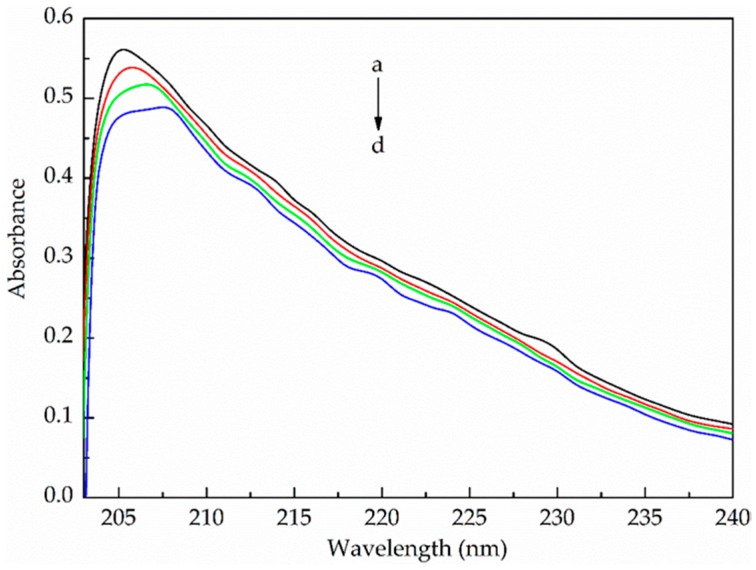
UV-Vis spectra of CAT with different concentrations of 2-MT (versus the same concentration of 2-MT solution). Conditions: CAT: 1.0 × 10^−7^ mol·L^−1^; 2-MT: 0 mol·L^−1^ (a); 1 × 10^−5^ mol·L^−1^ (b); 2 × 10^−5^ mol·L^−1^ (c); 3 × 10^−5^ mol·L^−1^ (d); pH 7.4; T = 291 K.

UV–Vis absorption spectroscopy is a widely used technique to explore the structural changes of proteins. The UV–Vis absorption spectra of CAT in the absence and presence of 2-MT is shown in Figure 5. Depending on its backbone conformation, the maximum absorption of CAT approximately appeared at 205 nm. However, with increasing 2-MT addition, the UV–Vis absorbance was gradually reduced, and the position of the absorption peak was red-shifted to roughly 208 nm. The dynamic changes implied that the interaction with 2-MT could lead to the loosening and unfolding of the CAT peptide chains [34]. Thus, the hydrophobicity of the CAT skeleton decreased.

#### 2.3.2. Synchronous Fluorescence Spectroscopy

Synchronous fluorescence spectroscopy can provide important information on the polarity changes of the microenvironments around molecular fluorophores [35]. The technology is based on simultaneous scanning of the excitation and emission monochromators while keeping a constant wavelength interval. When the wavelength interval (Δλ) is fixed at 15 or 60 nm, the synchronous fluorescence is characterized by tyrosine residues or tryptophan residues, respectively [36].

**Figure 6:**
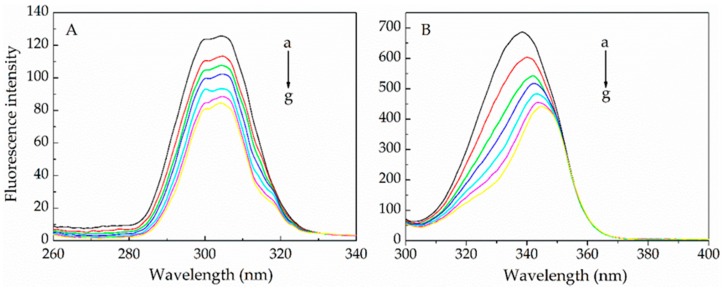
Synchronous fluorescence spectra of CAT with different concentrations of 2-MT. Conditions: (**A**) Δλ = 15 nm and (**B**) Δλ = 60 nm; CAT: 1.0 × 10^−7^ mol·L^−1^; 2-MT: 0 mol·L^−1^ (a); 1 × 10^−5^ mol·L^−1^ (b); 2 × 10^−5^ mol·L^−1^ (c); 3 × 10^−5^ mol·L^−1^ (d); 4 × 10^−5^ mol·L^−1^ (e); 5 × 10^−5^ mol·L^−1^ (f); 6 × 10^−5^ mol·L^−1^ (g); pH 7.4; T = 291 K.

The CAT synchronous fluorescence spectra with and without 2-MT addition is shown in Figure 6. When the Δλ was kept at 15 nm (Figure 6A), the variation of the 2-MT concentration did not obviously change the position of the emission peaks, which suggested that 2-MT had fewer effects on the microenvironment around the tyrosine residues of CAT. However, the position of the emission peak at Δλ = 60 nm was red-shifted from 338 nm to 345 nm as the 2-MT concentration increased from 0 to 6 × 10^−5^ mol·L^−1^ (Figure 6B). The results indicated that the CAT interaction with 2-MT could increase the exposure of its tryptophan residues to a more polar microenvironment [37], thereby further confirming the 2-MT impacts on CAT conformation.

#### 2.3.3. Circular Dichroism

**Figure 7:**
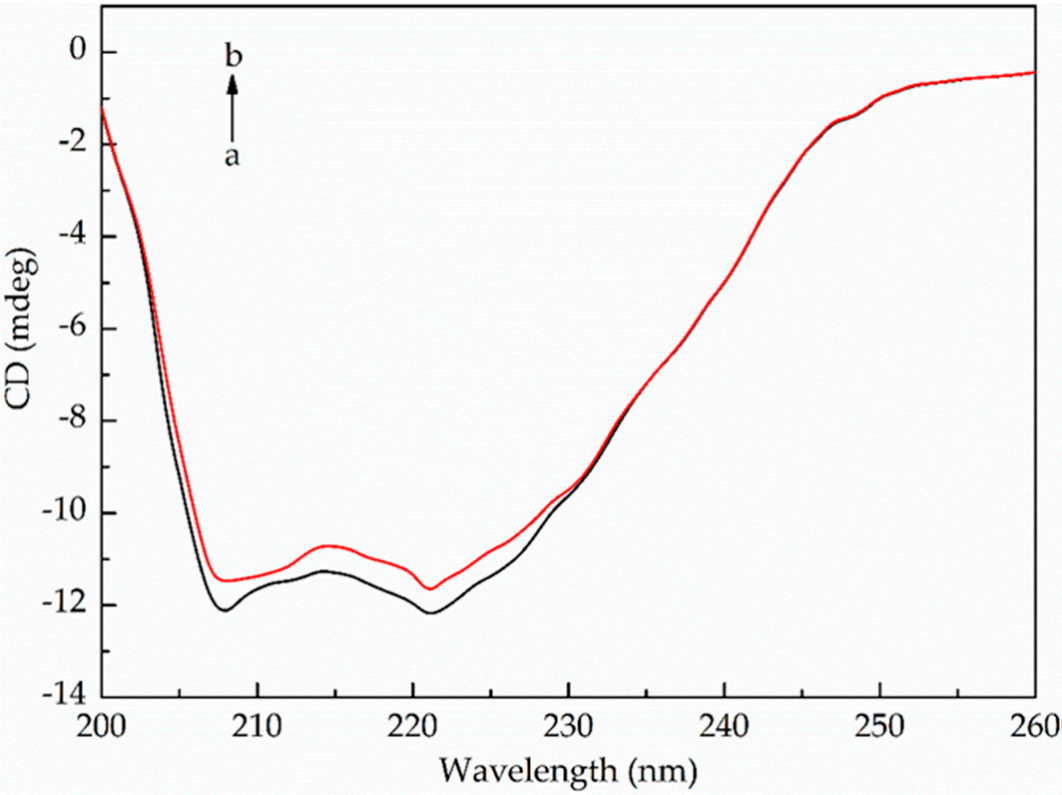
CD spectra of CAT in the absence and presence of 2-MT. Conditions: CAT: 1 × 10^−7^ mol·L^−1^; 2-MT: 0 mol·L^−1^ (a); 1 × 10^−6^ mol·L^−1^ (b); pH 7.4; T = 291 K.

The results above preliminarily indicated the CAT conformational changes are induced by 2-MT. In order to further investigate the 2-MT effects on the secondary structure of CAT, circular dichroism (CD) measurements were performed in the absence and presence of 2-MT. Shown in Figure 7, there were two negative peaks (at 208 and 221 nm) in the CD spectra of CAT, which is characteristic of the protein α-helix [38]. Subsequently, the CD spectra was analyzed using the CDPro software package, and the results are summarized in Table 3. It was shown that CAT contained the secondary structures of α-helix (19.7%), β-sheet (31.1%), turns (23.3%), and unordered (25.8%). However, the addition of 2-MT into the CAT solution (molar ratio: 10:1) reduced α-helix proportion by 0.7%. Meanwhile, the proportions of β-sheet and turns were respectively increased by 0.4% and 0.3%. The comparison demonstrated that 2-MT could cause some secondary structural changes in CAT. The decrease of α-helix proportion suggests a certain unfolding and denaturation of CAT induced by 2-MT [9], which is in accordance with the results from the UV–Vis absorption and synchronous fluorescence analysis.

### 2.4. Evaluation of 2-MT Inhibitory Effect on CAT Activity

**Figure 8:**
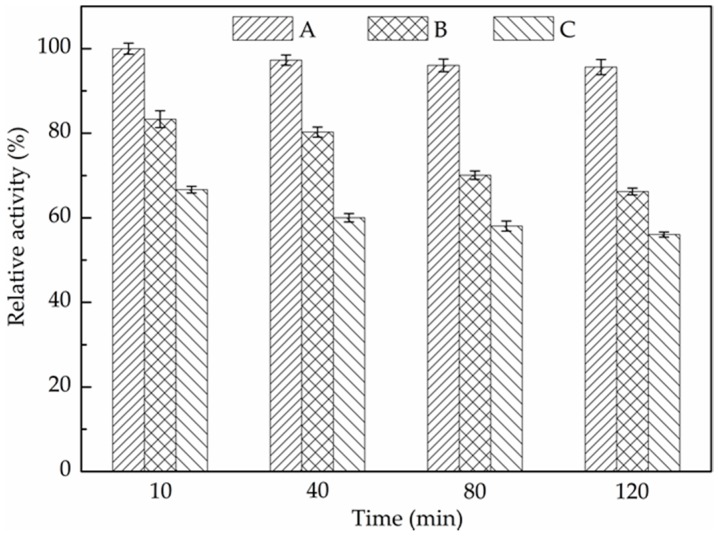
Inhibitory effect of 2-MT on CAT activity. Conditions: CAT: 1.0 × 10^−6^ mol·L^−1^; 2-MT: 0 mol·L^−1^ (A); 3 × 10^−5^ mol·L^−1^ (B); 1 × 10^−4^ mol·L^−1^ (C); pH 7.4, T = 291 K.

According to the experimental results above, it was found that 2-MT could induce CAT conformational changes by binding to the junctional region of the CAT subdomains, and the binding site was close to the enzyme active sites on chain A and chain D (Section 2.2 and Section 2.3). In order to verify the potential toxic effects of 2-MT caused by the interaction, the CAT activities under different 2-MT concentrations and exposure times were determined. As shown in Figure 8, since there was a strong interaction between 2-MT and CAT with a high binding constant (Section 2.1), the enzyme activity was immediately inhibited when exposed to 2-MT for just 10 min. The relative activities were significantly decreased to 83.3% and 66.7% when 2-MT concentrations were 3 × 10^−5^ mol·L^−1^ and 1 × 10^−4^ mol·L^−1^, respectively. In addition, the CAT activity gradually reduced as the exposure time increased, and the residual activity was only 56.1% with 2-MT inhibition (1 × 10^−4^ mol·L^−1^) for 120 min. Therefore, these experiments demonstrated that both 2-MT concentration and exposure time could influence the inhibition of CAT activity by 2-MT.

## 3. Materials and Methods

### 3.1. Reagents

Catalase (from bovine liver) was purchased from Sigma and was dissolved in ultrapure water to prepare stock solution (1.0 × 10^−5^ mol·L^−1^), which was then preserved at 0–4 °C. All the other chemicals were of analytical grade, and were purchased from Sinopharm Chemical Reagent Co., Ltd. The stock solution (1.0 × 10^−3^ mol·L^−1^) of 2-mercaptothiazoline (2-MT) was prepared. The reaction pH was controlled using 0.2 mol·L^−1^ phosphate buffer (mixture of NaH_2_PO_4_·2H_2_O and Na_2_HPO_4_·12H_2_O, pH 7.4). The NaCl stock solution (0.5 mol·L^−1^) was used to simulate the osmotic pressure in organisms. All solutions were prepared with ultrapure water (18.25 MΩ).

### 3.2. Apparatus and Measurements

#### 3.2.1. Fluorescence Measurements

The fluorescence measurements were conducted on a fluorescence spectrophotometer (RF-5301PC, Shimadzu, Kyoto, Japan) equipped with a 1 cm cell. The excitation wavelength was set at 280 nm, and the emission spectra were recorded in the range of 290–500 nm with a scan speed of 1200 nm/min and a photomultiplier tube (PMT) voltage of 700 V. The excitation and emission slit widths were both 5 nm.

#### 3.3.2. Molecular Docking Investigation

Docking calculations were performed on a CAT protein model (PDB code 1TGU) using AutoDock 4.2. As the predominant tautomer of 2-MT in solution [11], the thione form was used for the molecular docking. The 3D structure of 2-MT was downloaded from the ZINC Database (http://zink.docking.org/). With the aid of AutoDock, the ligand root of 2-MT was detected and the rotatable bonds were defined. In addition, H_2_O molecules were removed from the CAT model, while polar hydrogen atoms and Compute Gasteiger charges were added. Along the X-, Y-, and Z-axes with 0.375 Å grid spacing, the molecular docking was first performed with the grid size set to 126, 126 and 126 Å. On this basis, the grid size was subsequently shrunk to 86, 86 and 86 Å in order to improve the docking accuracy. Docking simulations were carried out by the Lamarckian genetic algorithm (LGA) search method. Each run of the docking experiment was set to terminate after a maximum of 250,000 energy evaluations, and the population size was set to 150. By the molecular docking simulation, the conformation with the lowest binding free energy was used for further analysis.

#### 3.3.3. UV–Visible Absorption Measurements

The UV–Visible absorption was measured using a double beam spectrophotometer (UV-6100, Mapada, Shanghai, China), with 1 cm quartz cells and a slit width of 2 nm. The wavelength range was set at 200–240 nm. The reference was the corresponding 2-MT solution without CAT addition.

#### 3.3.4. Synchronous Fluorescence Measurements

The synchronous fluorescence spectra of CAT with different 2-MT concentrations were measured (Δλ = 15 nm, λ_ex_ = 260–340 nm and Δλ = 60 nm, λ_ex_ = 300–400 nm, respectively) using a fluorescence spectrophotometer (RF-5301PC, Hitachi, Japan). The excitation and emission slit widths were set at 5 nm. The scan speed and PMT voltage were 1200 nm/min and 700 V, respectively.

#### 3.3.5. Circular Dichroism (CD) Measurements

The CD measurements were performed on a spectrophotometer (MOS-450/AF-CD, Bio-Logic, Claix, France). The CAT spectra in the absence and presence of 2-MT were recorded in the wavelength range of 200–260 nm, with a bandwidth of 4 nm and a scanning speed of 1 nm/2 s.

#### 3.3.6. CAT Activity Determination

The CAT catalyzes the decomposition of hydrogen peroxide into water and oxygen, and its activity can be assayed by monitoring the absorbency decrease of hydrogen peroxide at 240 nm [39]. The determination was carried out in a pH 7.4 phosphate buffer (20 mM) containing 9 mM hydrogen peroxide. The reaction was initiated by adding the CAT that was exposed to various doses of 2-MT for different times. The relative activity of CAT was calculated by the following equation:

(5) $$\text{Relative activity}=\frac{\Delta A_{1}}{\Delta A_{0}}\times 100\%$$

where ΔA_0_ represents the absorbency reduction (240 nm) in 2 min after adding CAT without any exposure to 2-MT; ΔA_1_ represents the absorbency reduction in 2 min after adding the CAT that was exposed to various doses of 2-MT for different time.

## 4. Conclusions

In this study, the toxicological interaction of 2-mercaptothiazoline (2-MT) with the pivotal antioxidant enzyme—catalase (CAT) was investigated at molecular level by multiple spectroscopic techniques and molecular modeling. 2-MT could quench the CAT fluorescence via a static quenching mechanism through formation of the 2-MT/CAT complex. Van der Waals’ forces and hydrogen bonding were primarily responsible for the interaction of 2-MT with CAT at one binding site. The molecular docking simulation indicated that 2-MT could bind to the junctional region of the CAT subdomains, and the binding site was close to the CAT active sites on chain A and chain D. The interaction had few effects on the microenvironment around the tyrosine residues of CAT, but increased the exposure of tryptophan residues to a more polar microenvironment. The presence of 2-MT also induced secondary structural changes, and caused a certain unfolding and denaturation in CAT. Due to the molecular binding, the activity of CAT was significantly inhibited by 2-MT, where 2-MT concentration and exposure time both affected the inhibitory action. Taken together, this work provides valuable insight into the interaction between 2-MT and CAT at the molecular level, and helps us to understand the mechanism of 2-MT toxicity on the environment and human health.

## Figures and Tables

**Table 1:** Stern–Volmer quenching constants for the interaction of 2-MT with CAT under different temperatures (291 K, 300 K, 309 K, and 318 K).

pH	T (K)	*K*_SV_ (×10^4^ L·mol^−1^·s^−1^)	*k*_q_ (×10^12^ L·mol^−1^·s^−1^)	*R* ^1^	S.D. ^2^
7.4	291	1.59	1.59	0.9935	0.0429
7.4	300	1.46	1.46	0.9939	0.0384
7.4	309	1.35	1.35	0.9922	0.0398
7.4	318	1.23	1.23	0.9910	0.0391

^1^ *R* is the correlation coefficient; ^2^ S.D. is the standard deviation for the *K*_SV_ values.

**Table 2:** Binding constants and thermodynamic parameters from 2-MT/CAT interaction (pH 7.4).

*T* (K)	*K*_a_ (× 10^4^ mol·L^−1^)	*n*	*R* ^1^	Δ*H*° (kJ·mol^−1^)	Δ*S*° (J·mol^−1^·K^−1^)	Δ*G*° (kJ·mol^−1^)
291	2.97	1.14	0.9987	−80.79	−192.26	−24.84
300	1.07	1.07	0.9973			−23.11
309	0.41	0.96	0.9955			−21.38
318	0.17	0.83	0.9956			−19.65

^1^ *R* is the correlation coefficient.

**Table 3:** Effects of 2-MT on the proportion of secondary structural elements in CAT at 291 K.

Molar Ratio of CAT to 2-MT	Secondary Structural Elements in CAT			
	α-Helix (%)	β-Sheet (%)	Turns (%)	Unordered (%)
1:0	19.7	31.1	23.3	25.8
1:10	19.0	31.5	23.6	25.9

## References

[1] Gaetani G.F., Ferraris A.M., Rolfo M., Mangerini R., Arena S., Kirkman H.N. (1996). Predominant role of catalase in the disposal of hydrogen peroxide within human erythrocytes. Blood.

[2] Sofo A., Scopa A., Nuzzaci M., Vitti A. (2015). Ascorbate peroxidase and catalase activities and their genetic regulation in plants subjected to drought and salinity stresses. Int. J. Mol. Sci..

[3] Giustarini D., Dalledonne I., Tsikas D., Rossi R. (2009). Oxidative stress and human diseases: Origin, link, measurement, mechanisms, and biomarkers. Crit. Rev. Clin. Lab. Sci..

[4] Gonenc A., Hacisevki A., Aslan S., Torun M., Simsek B. (2012). Increased oxidative DNA damage and impaired antioxidant defense system in patients with gastrointestinal cancer. Eur. J. Intern. Med..

[5] Noguer M.A., Cerezo A.B., Navarro E.D., Garcia-Parrilla M.C. (2012). Intake of alcohol-free red wine modulates antioxidant enzyme activities in a human intervention study. Pharmacol. Res..

[6] Jan A.T., Azam M., Siddiqui K., Ali A., Choi I., Haq Q.M.R. (2015). Heavy metals and human health: Mechanistic insight into toxicity and counter defense system of antioxidants. Int. J. Mol. Sci..

[7] Wu Y.H. (2007). Study on the interaction between salicylic acid and catalase by spectroscopic methods. J. Pharm. Biomed..

[8] Chi Z.X., Liu R.T., Zhang H. (2010). Potential enzyme toxicity of oxytetracycline to catalase. Sci. Total Environ..

[9] Cao Z.Z., Liu R.T., Yang B.J. (2013). Potential toxicity of sarafloxacin to catalase: Spectroscopic, ITC and molecular docking descriptions. Spectrochim. Acta A.

[10] Park Y., Yang S., Lee M., Lim H., Kim Y., Kim S., Lee H. (2013). Confirmation of the coexistence of two tautomers of 2-mercaptothiazoline on the Ge(100) surface. Phys. Chem. Chem. Phys..

[11] Rabie U.M., Abou-El-Wafa M.H.M., Nassar H. (2011). Multiple and sequential charge transfer interactions occurring in situ: A redox reaction of thiazolidine-2-thione with 2,3-dichloro-5,6-dicyano-1,4-benzoquinone. Spectrochim. Acta A.

[12] Abbehausen C., Paiva R.E.F.D., Formiga A.L.B., Corbi P.P. (2012). Studies of the tautomeric equilibrium of 1,3-thiazolidine-2-thione: Theoretical and experimental approaches. Chem. Phys..

[13] Chen Y.H., Chang C.Y., Chen C.C., Chiu C.Y., Yu Y.H., Chiang P.C., Chang C.F., Shie J.L. (2004). Kinetics of ozonation of 2-Mercaptothiazoline in an electroplating solution. Ind. Eng. Chem. Res..

[14] Solmaz R., Kardas G., Culha M., Yazici B., Erbil M. (2008). Investigation of adsorption and inhibitive effect of 2-mercaptothiazoline on corrosion of mild steel in hydrochloric acid media. Electrochim. Acta.

[15] Pérez-Quintanilla D., Hierro I.D., Fajardo M., Sierra I. (2006). 2-Mercaptothiazoline modified mesoporous silica for mercury removal from aqueous media. J. Hazard. Mater..

[16] Rabie U.M., Abou-El-Wafa M.H.M., Nassar H. (2011). In vitro simulation of the chemical scenario of the action of an anti-thyroid drug: Charge transfer interaction of thiazolidine-2-thione with iodine. Spectrochim. Acta A.

[17] Chang C.F., Chang C.Y., Holl W. (2004). Investigating the adsorption of 2-mercaptothiazoline on activated carbon from aqueous systems. J. Colloid Interface Sci..

[18] Chen Y.H., Chang C.Y., Chen C.C., Chiu C.Y. (2006). Kinetics of ozonation of 2-Mercaptothiazoline in an electroplating solution combined with UV radiation. Ind. Eng. Chem. Res..

[19] Chen Y.H., Chang C.Y., Chen C.C., Chiu C.Y., Yu Y.H., Chiang P.C., Ku Y., Chen J.N., Chang C.F. (2004). Decomposition of 2-mercaptothiazoline in aqueous solution by ozonation. Chemosphere.

[20] Teng Y., Wang X., Zou L., Huang M., Du X. (2015). Experimental and theoretical study on the binding of 2-mercaptothiazoline to bovine serum albumin. J. Lumin..

[21] Thomes J.C., Comby F., Lagorce J.F., Buxeraud J., Raby J. (1992). Sites of action of 2-thiazoline-2-thiol on biogenesis of thyroid hormones. Jpn. J. Pharmacol..

[22] Papadopoulou A., Green R.J., Frazier R.A. (2005). Interaction of flavonoids with bovine serum albumin: A fluorescence quenching study. J. Agric. Food Chem..

[23] Vercillo N.C., Herald K.J., Fox J.M., Der B.S., Dattebaum J.D. (2007). Analysis of ligand binding to a ribose biosensor using site-directed mutagenesis and fluorescence spectroscopy. Protein Sci..

[24] Hamdi O.A., Feroz S.R., Shilpi J.A., Anouar E.H., Mukarram A.K., Mohamad S.B., Tayyab S., Awang K. (2015). Spectrofluorometric and molecular docking studies on the binding of curcumenol and curcumenone to human serum albumin. Int. J. Mol. Sci..

[25] Zhang Y.Z., Zhou B., Zhang X.P., Huang P., Li C.H., Liu Y. (2009). Interaction of malachite green with bovine serum albumin: Determination of the binding mechanism and binding site by spectroscopic methods. J. Hazard. Mater..

[26] Cui F.L., Fan J., Li J.P., Hu Z.D. (2004). Interactions between 1-benzoyl-4-p-chlorophenyl thiosemicarbazide and serum albumin: Investigation by fluorescence spectroscopy. Bioorgan. Med. Chem..

[27] Lakowicz J.R., Weber G. (1973). Quenching of protein fluorescence by oxygen. Detection of structural fluctuations in proteins on the nanosecond time scale. Biochemistry.

[28] Chi Z.X., Liu R.T., Yang B.J., Zhang H. (2010). Toxic interaction mechanism between oxytetracycline and bovine hemoglobin. J. Hazard. Mater..

[29] Liu X.H., Xi P.X., Chen F.J., Xu Z.H., Zeng Z.Z. (2008). Spectroscopic studies on binding of 1-phenyl-3-(coumarin-6-yl)sulfonylurea to bovine serum albumin. J. Photochnol. Photobiol. B.

[30] Paramaguru G., Kathiravan A., Selvaraj S., Venuvanalingam P., Renganathan R. (2010). Interaction of anthraquinone dyes with lysozyme: Evidences from spectroscopic and docking studies. J. Hazard. Mater..

[31] Ross P.D., Subramanian S. (1981). Thermodynamics of protein association reactions: Forces contributing to stability. Biochemistry.

[32] Khan S.N., Islam B., Yennamalli R., Sultan A., Subbarao N., Khan A.U. (2008). Interaction of mitoxantrone with human serum albumin: Spectroscopic and molecular modeling studies. Eur. J. Pharm. Sci..

[33] Xu H., Yao N., Xu H., Wang T., Li G., Li Z. (2013). Characterization of the interaction between eupatorin and bovine serum albumin by spectroscopic and molecular modeling methods. Int. J. Mol. Sci..

[34] Yang Q.Q., Liang J.G., Han H.Y. (2009). Probing the Interaction of Magnetic Iron Oxide Nanoparticles with Bovine Serum Albumin by Spectroscopic Techniques. J. Phys. Chem. B.

[35] Teng Y., Ji F.Y., Li C., Yu Z.H., Liu R.T. (2011). Interaction mechanism between 4-aminoantipyrine and the enzyme lysozyme. J. Lumin..

[36] Guo M., Lu W.J., Li M.H., Wang W. (2008). Study on the binding interaction between carnitine optical isomer and bovine serum albumin. Eur. J. Med. Chem..

[37] Zhu J.F., Zhang X., Li D.J., Jin J. (2007). Probing the binding of flavonoids to catalase by molecular spectroscopy. J. Mol. Struct..

[38] Lu J.Q., Jin F., Sun T.Q., Zhou X.W. (2007). Multi-spectroscopic study on interaction of bovine serum albumin with lomefloxacin-copper(II) complex. Int. J. Biol. Macromol..

[39] Hideyuki K., Kazuaki Y., Hidetoshi M., Isao Y. (2012). Characterization of catalase from psychrotolerant psychrobacter piscatorii T-3 exhibiting high catalase activity. Int. J. Mol. Sci..

